# Risk of malignancy in patients with chronic kidney disease

**DOI:** 10.1371/journal.pone.0272910

**Published:** 2022-08-17

**Authors:** Ketki K. Tendulkar, Brendan Cope, Jianghu Dong, Troy J. Plumb, W. Scott Campbell, Apar Kishor Ganti

**Affiliations:** 1 Division of Nephrology, Department of Internal Medicine, University of Nebraska Medical Center, Omaha, NE, United States of America; 2 Division of Rheumatology, Department of Internal Medicine, University of Nebraska Medical Center, Omaha, NE, United States of America; 3 Department of Biostatistics, College of Public Health, University of Nebraska Medical Center, Omaha, NE, United States of America; 4 Department of Pathology/Microbiology, University of Nebraska Medical Center, Omaha, NE, United States of America; 5 Division of Hematology and Oncology, Department of Internal Medicine, VA Nebraska-Western Iowa Health Care System and University of Nebraska Medical Center, Omaha, NE, United States of America; Faculty of Medicine, Saint-Joseph University, LEBANON

## Abstract

**Background:**

Fifteen percent of US adults have chronic kidney disease (CKD). The effect of CKD on the development of different malignancies is unknown. Understanding the effect of CKD on the risk of development of cancer could have important implications for screening and early detection of cancer in these patients.

**Methods:**

Adult CKD patients [estimated GFR (eGFR) <60ml/min/1.73m^2^] between January 2001 and December 2020 were identified in this single institution study. Patients were divided into four stages of CKD by eGFR. The incidence of cancer and time to development of the first cancer were identified. Multivariable models were used to compare the overall cancer incidence while considering death as a competing risk event and adjusting for relevant covariates (sex, race, diabetes, hypertension, CAD, smoking or not, BMI, and CKD stages). Separate multivariable models of the incidence of cancers were conducted in each age group. Multivariable Cox models were used to fit the overall death adjusting for relevant covariates. Patients were censored at the conclusion of the study period (December 31, 2020). Statistical analysis was performed with SAS software (version 9.4).

**Results:**

Of the 13,750 patients with a diagnosis of CKD in this cohort, 2,758 (20.1%) developed a malignancy. The median time to development of cancer following a diagnosis of CKD was 8.5 years. Factors associated with the risk of developing cancer in CKD patients included increasing age, male sex and worsening chronic kidney disease, while diabetes was associated with a lower risk of malignancy. On multivariate analysis, the factors associated with increased mortality in patients who developed cancer included increasing age, diabetes and lower eGFR.

**Conclusion:**

CKD is an increased risk factor for the development of various malignancies. Age appropriate cancer screening should be aggressively pursued in those with progressive CKD.

## Introduction

Kidney disease is a significant cause of morbidity and mortality in the United States, with an estimated 30 million people (15% of US adults) living with Chronic Kidney disease (CKD) in 2017 [https://www.cdc.gov/kidneydisease/kidney_factsheet.pdf]. Similarly, cancer is a leading cause of morbidity and mortality in the United States. People with end stage kidney disease (ESKD) are at increased risk for at least certain types of cancers [1, 2]. However, it is uncertain if this increased risk begins earlier in the spectrum of chronic kidney disease (CKD). The effect of CKD may vary for different types of cancer and ages [3]. The link between cancer and CKD appears to be bi-directional; cancer can cause CKD either directly or indirectly through the adverse effects of therapies, CKD may conversely be a risk factor for cancer [4].

Community-based reports evaluating the association between estimated glomerular filtration rate (eGFR) and the risk of cancer are conflicting. A Scandinavian population-based study has explored plausible links between kidney function and cancer incidence [5]. They found a slightly higher risk of skin and urogenital cancer in patients with mild to severe CKD. Yang et al. stressed that CKD was prevalent in almost a third of patients (32.4%) with a new diagnosis of cancer [6]. Hence determining whether there is an association between the presence and severity of CKD with subsequent cancer risk and specifically examining if level of kidney function is differentially associated with specific cancer types could have important public health implications for screening and early detection of cancer in patients with CKD [7].

It is also important to determine the strength of this association as detection of cancer in patients with CKD will influence not only their short-term outcomes but also options for adequate therapy of the underlying malignancy [8]. Studies have shown an increased risk of death in cancer patients with CKD as compared to those without CKD [9].

However, only a few studies exploring the association between mild to severe chronic kidney disease and cancer have been done in the United States. Hence, we conducted a retrospective single institution cohort study of patients with chronic kidney disease to evaluate the association between non-dialysis-dependent CKD and the risk for non-skin cancers.

## Patients and methods

### Data source

This is a retrospective study of medical records from a fully de-identified data warehouse/registry operating under the UNMC IRB # 0132-14-EP. Our cohort was created by identifying adult CKD patients between January 2001 and December 2020 from the Clinical Research Analytics Environment (CRANE) database. CRANE is supported by funding from the National Institute of General Medical Sciences, U54 GM115458 and the Patient Centered Outcomes Research Institute, PCORI CDRN-1306-04631. The content is solely the responsibility of the authors and does not necessarily represent the official views of the NIH or PCORI.

The CRANE database is based on the i2b2 (https://www.i2b2.org/) data structure and populated with data extracted using extra, transform, load (ETL) processes from the Nebraska Medicine/University of Nebraska Medical Center electronic health record at regular interval consistent with methods broadly deployed in PCORnet data repositories (https://gpctr.unmc.edu/cores/biomedical-informatics-cyberinfrastructure/crane/). Data specific to diagnoses are represented by ICD-9-CM (pre-October 2015) and ICD-10-CM (post October 2015) for all patient/physician encounter, SNOMED CT (Systematized Nomenclature for Medicine–Clinical Terms) for running patient problem lists and patient medical history. Laboratory data is encoded using LOINC (Logical Observations Identifiers Names and Codes) terms. Demographic information is represented using PCORnet defined classifications for age, sex, race and ethnicity. The CRANE database contains de-identified, longitudinal health data for patients who received care at our institution. The CRANE database serves as the primary data source for our participation in the national Patient-Centered Outcomes Research Institute (PCORI) [10] and the National COVID Cohort Collaborative (N3C) [11]. CRANE and related studies include targeted genomic cancer treatment evaluations [12] (3), Emergency Department presentations [13] and multiple N3C studies [14–16].

### Cohort description

The cohort consisted of all patients with chronic kidney disease and the outcome was the development of cancer and overall survival. Patients were considered to have CKD if their estimated glomerular filtration rate was less than 60 ml/min/1.73m^2^, or if they had persistent albuminuria, proteinuria or structural abnormalities on imaging studies based on KDIGO guidelines. Patients were then further divided by their range of eGFR per MDRD equation into four groups as follows: > = 60 ml/min, 45–59 ml/min, 30–44 ml/min and <30 ml/min/1.73m^2^. The study baseline date is the first date that the patient met the criteria for chronic kidney disease based on either an eGFR or albuminuria or structural abnormalities on imaging studies.

The patient cohort consisted of adult patients with a diagnosis of CKD as identified by ICD9/ICD10/SNOMED code. (S1 Table in S1 File) Patients diagnosed with End Stage Kidney Disease (ESKD) prior to a diagnosis of cancer, pregnant patients, and patients who received a kidney transplant were excluded. (S2 Table in S1 File) Data pertaining to each patient in the cohort included diagnostic status of hypertension, diabetes, Chronic Obstructive Pulmonary Disease (COPD), and coronary artery disease (CAD), dates of diagnosis, estimated glomerular filtration rate (eGFR), sex, age, BMI and smoking status. (S3 Table in S1 File)

Of all these patients in our cohort, we investigated whether they acquired their first cancer diagnosis over the follow-up period of 20 years. We grouped all patients into several individual groups by age at the CKD diagnosis. Among patients who had a cancer during this period, we also investigated patient survival outcomes.

### Statistical methods

Categorical data were summarized using counts and percentages and continuous variables were reported as median (interquartile range) or mean ± standard deviation as appropriate. Patient demographics and characteristics across groups were compared using analysis of variance or nonparametric statistics (Kruskal-Wallis tests) and chi-squared tests as appropriate. The cumulative incidence method and Gray’s tests were used to compare patient cancer curves in individual CKD groups by the age at CKD diagnosis.

Multivariable competing risk models were used to compare the overall cancer incidence while considering death as a competing risk event and adjusting for relevant covariates, which included age, sex, race, diabetes, hypertension, CAD, smoking history, BMI, and CKD stages. Separate multivariable models of the incidence of cancers were conducted in each age group after adjusting for sex, race, diabetes, hypertension, CAD, smoking history, BMI, and CKD stages. Multivariable Cox models were used to fit the overall death adjusting for relevant covariates, which included age, sex, race, diabetes, hypertension, CAD, smoking history, BMI, and CKD stages. Patients were censored at the conclusion of the study period (December 31, 2020). Statistical analysis was performed with SAS software (version 9.4).

## Results

**Fig 1:**
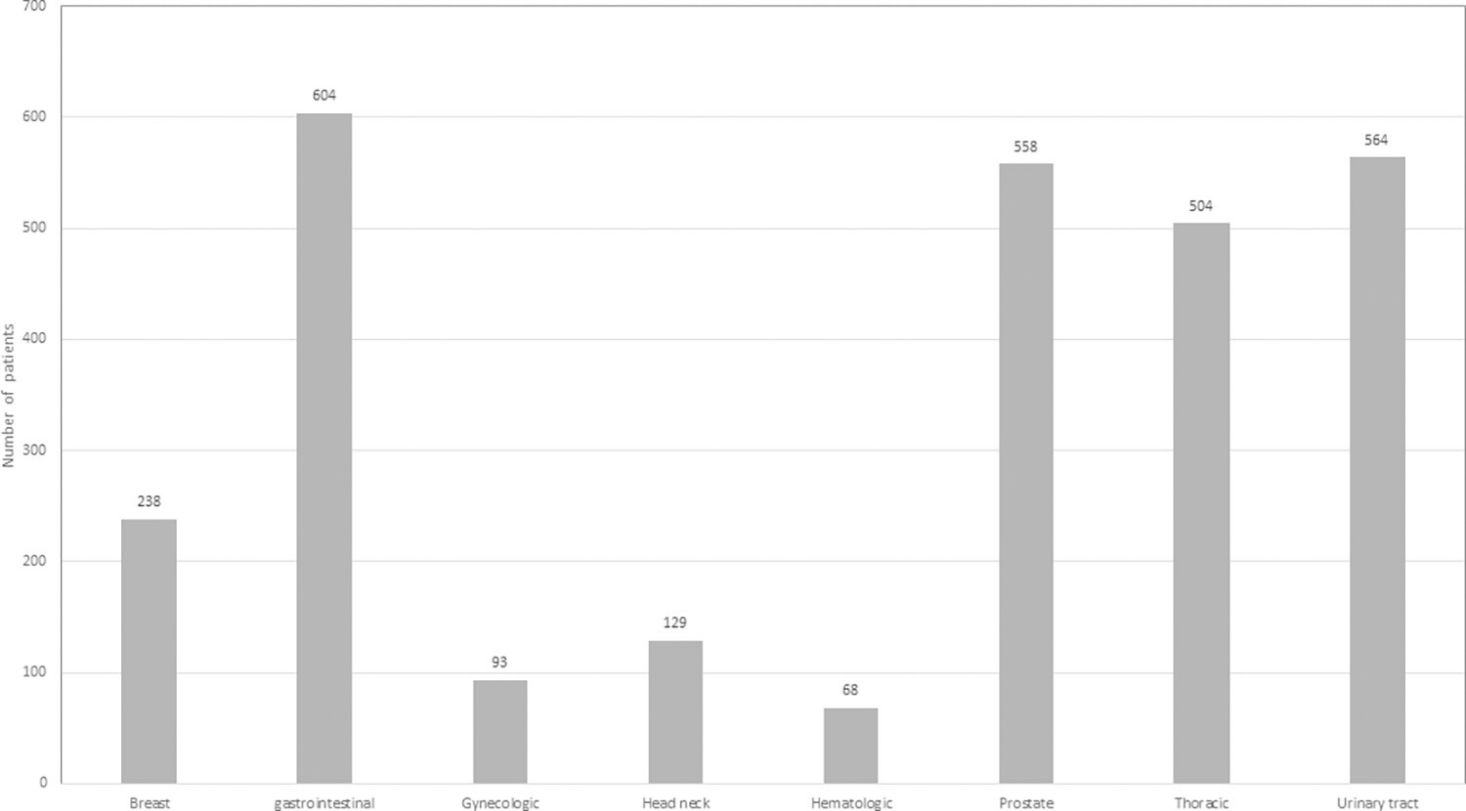
Summary of patients with a malignancy.

**Fig 2:**
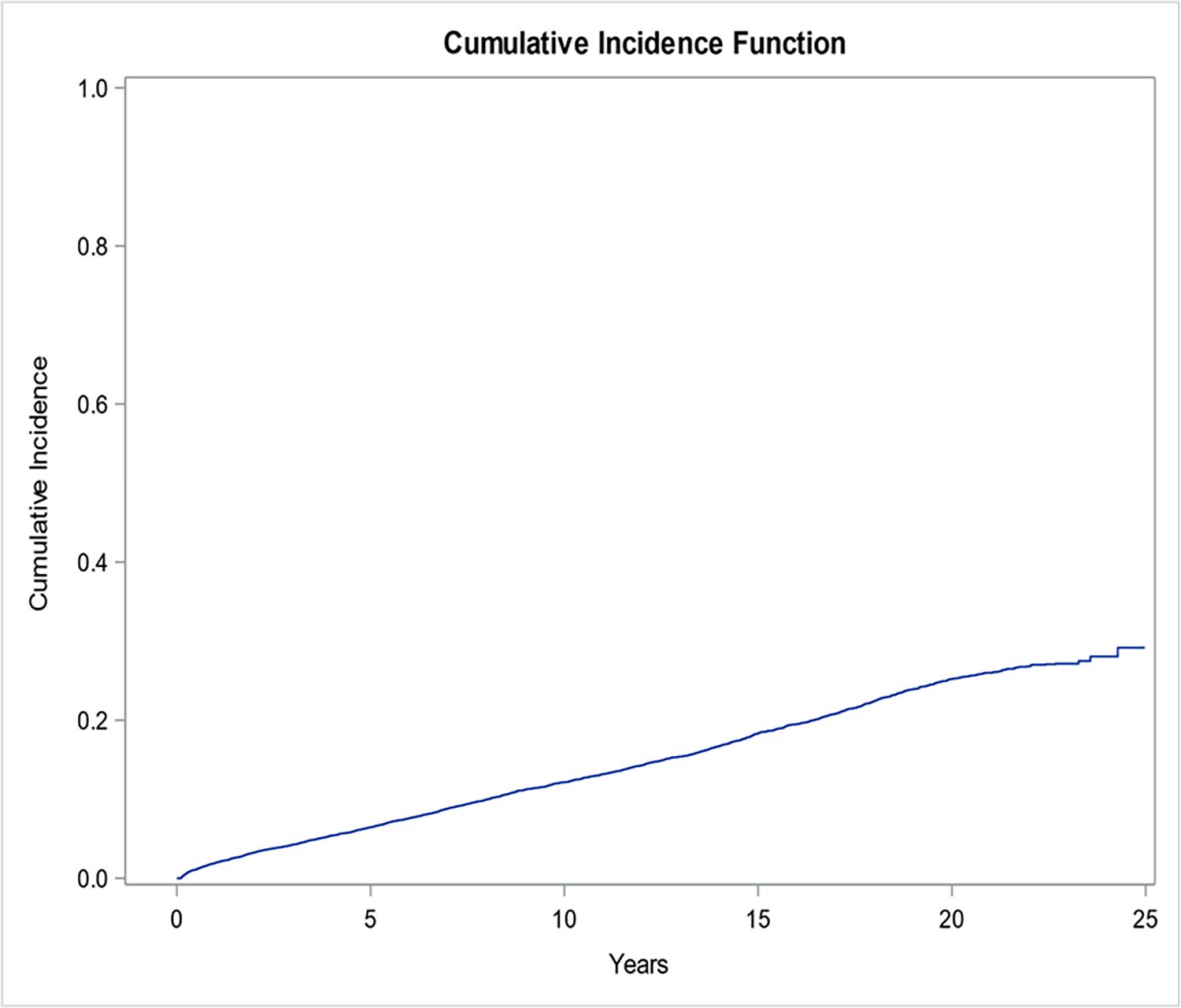
Cumulative incidence of overall malignancy.

We identified a total of 13,750 patients with a diagnosis of chronic kidney disease. The demographics of these patients are shown in Table 1. Of these, over a follow-up period of 20 years, 2,758 patients (20.1%) developed a cancer at a median age of 71 years (IQR: 63, 79 years). The most common cancers diagnosed were gastrointestinal malignancies (n = 604), prostate (n = 558), other urinary tract malignancies (n = 564), thoracic cancers (n = 504), breast cancer (n = 238), head and neck cancer (n = 129), gynecologic malignancies (n = 93) and hematologic malignancies (n = 68) (Fig 1). The cumulative incidence of development of any malignancy is depicted in Fig 2.

**Table 1:** Patient characteristic summary by stage of CKD.

	All cohort (N = 13,750)	> = 60 (N = 282)	45–59 (N = 8,197)	30–44 (N = 2,940)	<30 (N = 2,331)	P value
Male (%)	53	54	54	49	53	<0.001
Race (%)						<0.001
White	80.5	80.5	79.5	82.9	81.7	
AA Black	13.6	14.9	15.6	11.3	10.0	
Hispanic	2.4	1.4	2.1	2.3	3.4	
Others	3.5	4.2	2.8	3.5	4.9	
Age (%)						<0.001
<50	22.5	28.7	23.0	17.1	27.2	
50–65	33.5	50.7	36.5	29.1	26.4	
66–80	29.1	15.9	28.0	33.5	29.4	
>80	14.9	4.6	12.5	20.3	17.0	
Diabetes (%)	48.3	42.8	55.3	45.5	45.3	<0.001
Hypertension (%)	99.7	99.9	99.9	99.8	99.7	0.009
CAD (%)	36.9	30.1	39.1	39.6	27.7	<0.001
Smoking history (%)	50.0	49.5	49.7	48.6	53.6	<0.001
BMI (%)						<0.001
<18.5	8.3	3.9	4.7	9.0	20.8	
18.5–24.9	20.0	17.4	19.4	21.0	21.3	
25.0–29.9	27.1	29.9	28.6	27.1	21.4	
> = 30	44.5	48.7	47.3	42.9	26.5	

The median age (IQR) at diagnosis of each individual group of cancer was: gastrointestinal malignancies [69 years (61, 78)], prostate [74 years (67, 79)], other urinary tract malignancies [69 years (60, 78)], thoracic cancers [71 years (64, 79)], breast cancer [71 years (64, 79)], head and neck cancer [67 years (58, 76)], gynecologic malignancies [68 years (60, 75)], and hematologic malignancies [70 years (55, 77)].

The median time to development of cancer following a diagnosis of CKD was 8.50 years. When patients were classified based on age of diagnosis of CKD, the median ages (IQR) of their cancer diagnosis were as follows: <50: 54 years (46, 60); 50–65: 65 years (56–80); 66–80: 76 years (72, 80); and > 80: 84 years (81, 88).

Of the 2868 patients who had a diagnosis of CKD below age 50 years, 406 (14.2%) developed a malignancy at a median duration of 11.05 years (IQR; 4.79, 16.07) after the CKD diagnosis. In patients diagnosed with CKD between the ages of 50–65 years, 22.2% developed a cancer at the median 6.87 years (IQR: 1.16, 13.27). Similarly, 22.2% of patients diagnosed with CKD between the ages of 66 and 80 years developed a malignancy at a median 1.86 years following the CKD diagnosis. Of the patients older than 80 years at the time of CKD diagnosis, 19.1% developed cancer at a median duration of 0.75 years (IQR: 0, 4.01) after they were diagnosed with chronic kidney disease.

**Fig 3:**
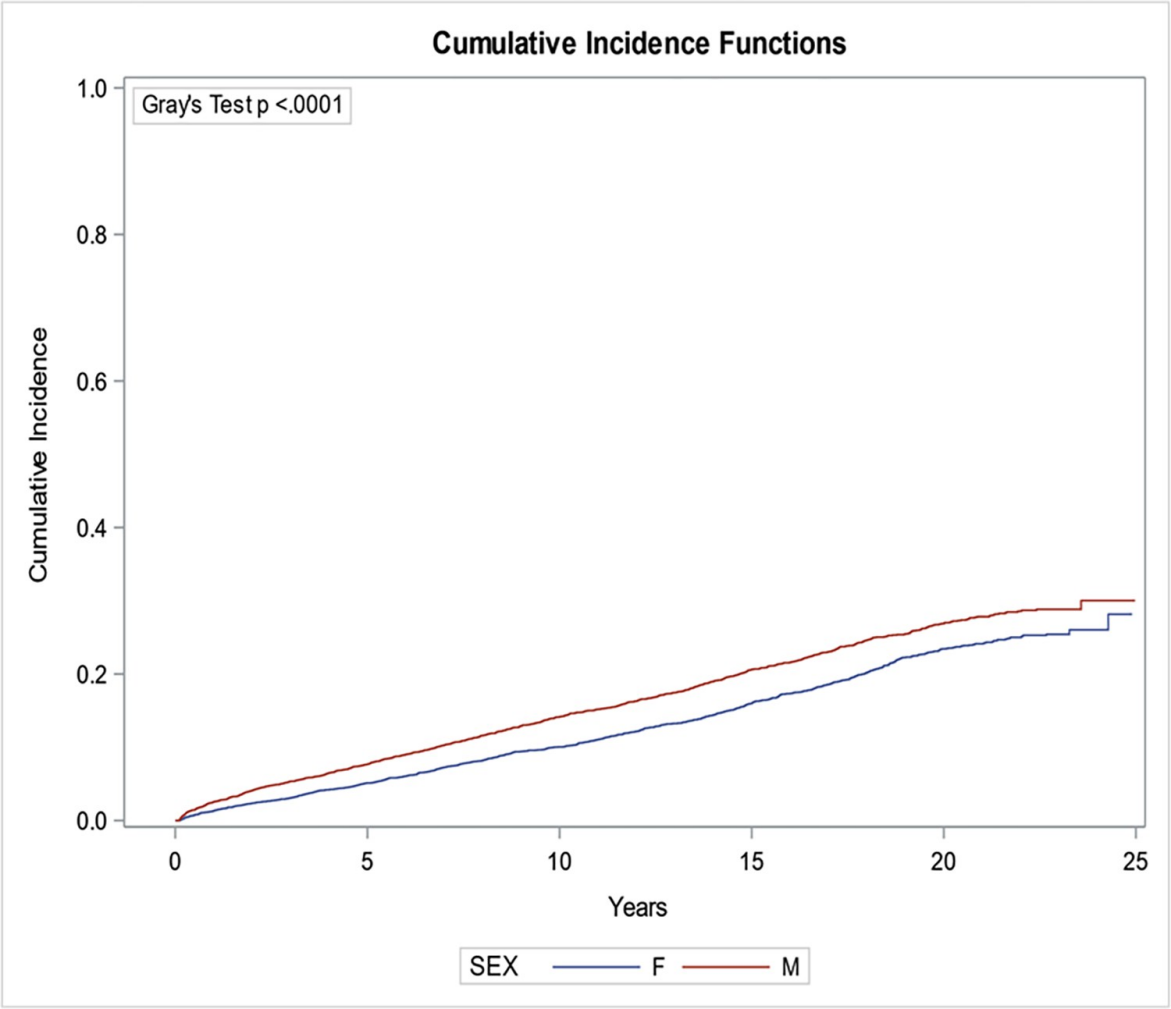
The cumulative incidence of overall malignancy by sex.

**Fig 4:**
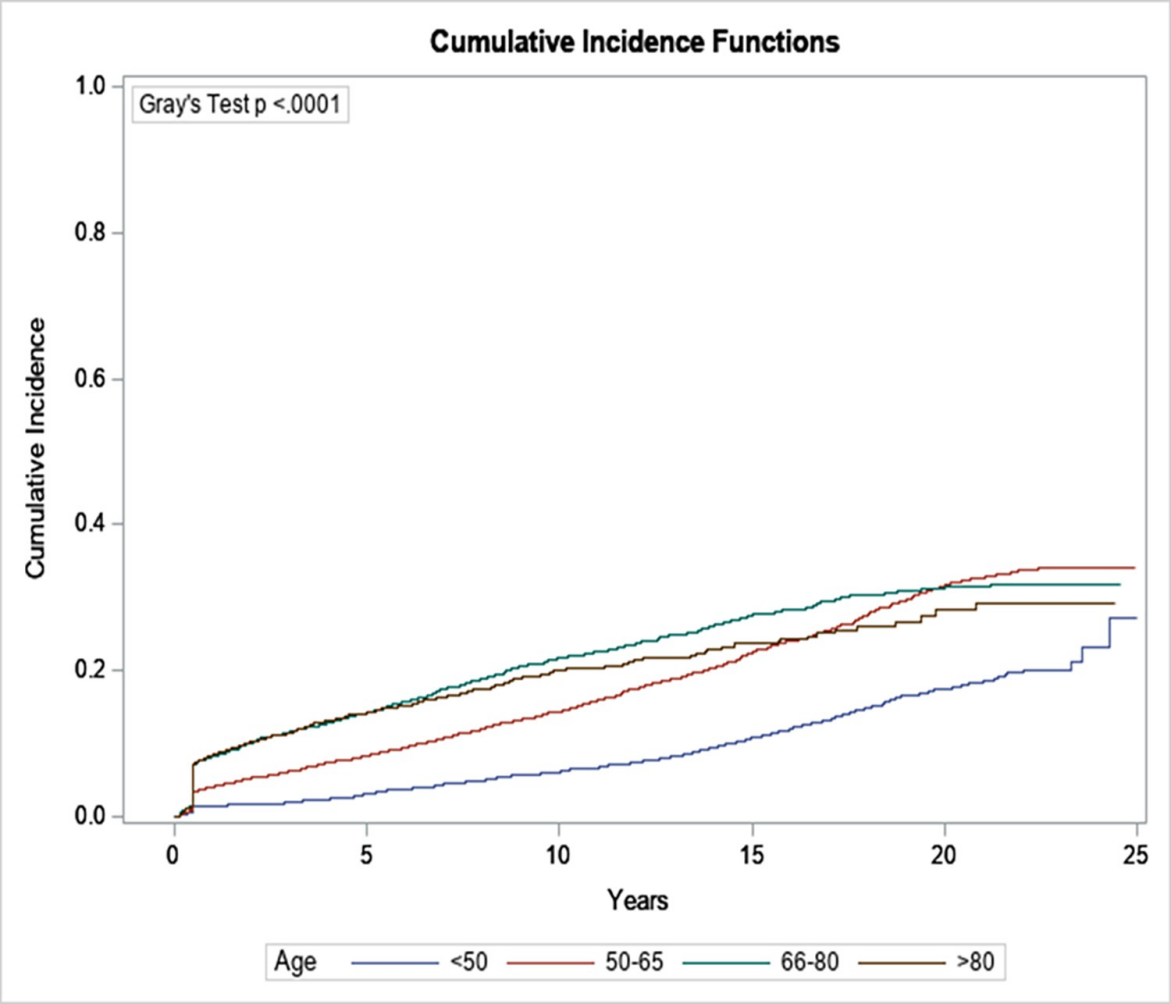
The cumulative incidence of overall malignancy by age.

**Fig 5:**
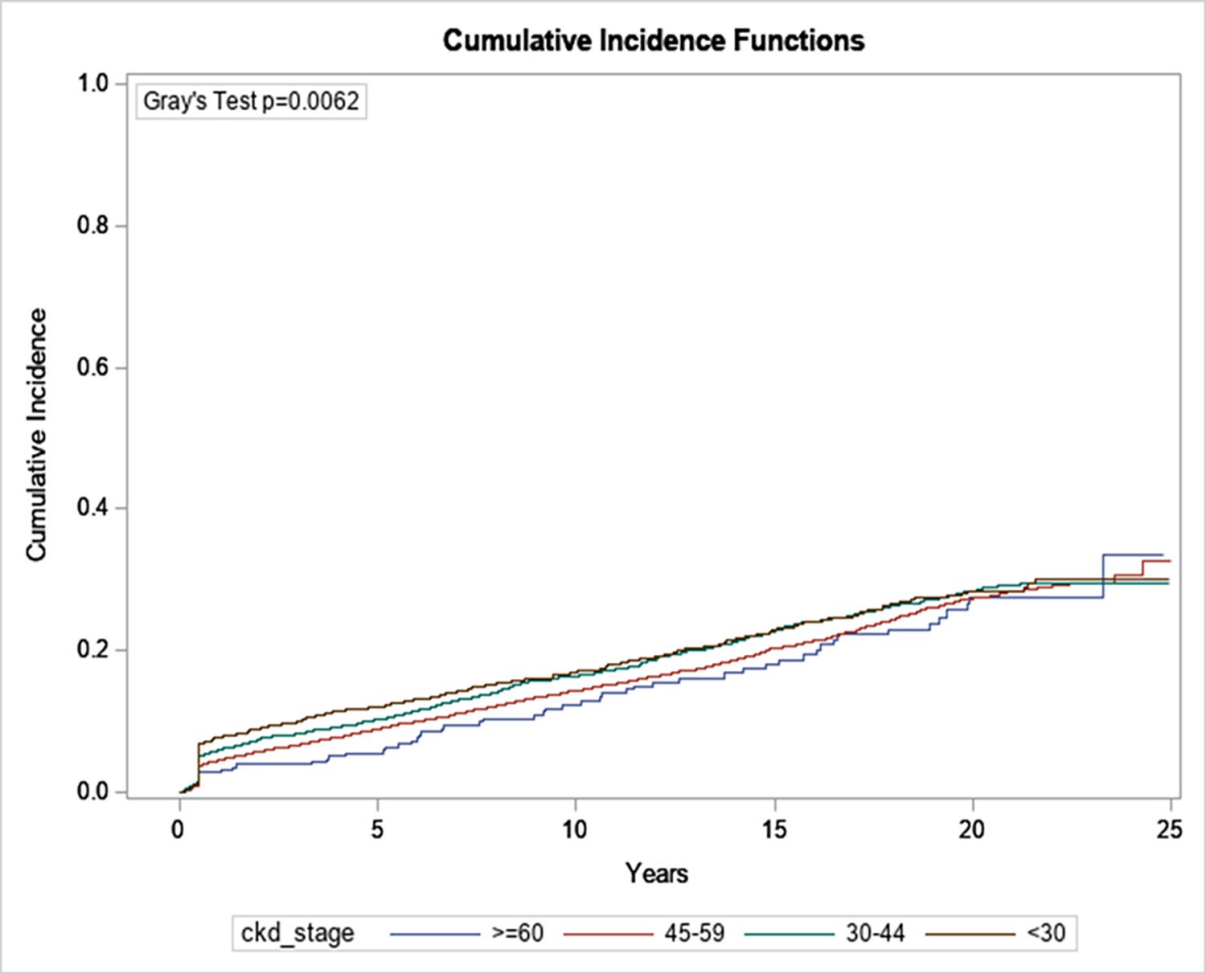
The cumulative incidence of overall malignancy by CKD stage.

Factors associated with the risk of developing cancer in CKD patients included male sex (Fig 3), increasing age (Fig 4) and worsening chronic kidney disease (Fig 5). Compared to patients <50 years of age, patients ≥ 80 were more likely to develop malignancy (HR: 3.34; 95% CI: 2.89, 3.88). Males had a higher risk of developing cancer as compared to female patients (HR: 1.44; 95% CI 1.33, 1.56). Similarly, patients who had a baseline eGFR of less than 30 ml/min/1.73m^2^ were 1.5 times more likely to develop malignancy than patients who had an eGFR of greater than 60 ml/min/1.73m^2^ (HR: 1.44; 95% CI: 1.08, 1.91) (Table 2). Interestingly the presence of diabetes was associated with a lower risk of malignancy (HR: 0.9; 95% CI: 0.83, 0.98).

**Table 2:** Factors affecting the development of malignancy after CKD diagnosis.

	Hazard Ratio (95% CI)	P value
Age		
<50	1.00	
50–65	2.23 (1.98, 2.51)	<0.001
66–80	3.15 (2.78, 3.56)	<0.001
>80	3.34 (2.89, 3.88)	<0.001
Male	1.44 (1.33, 1.56)	<0.001
Race		
White	1.00	
AA Black	0.92 (0.82, 1.03)	0.161
Hispanic	0.84 (0.65, 1.08)	0.193
Others	1.17 (0.96, 1.42)	0.109
Diabetes	0.90 (0.83, 0.98)	0.003
CAD	0.85 (0.80, 1.01)	0.051
Smoking history	1.18 (1.09, 1.28)	<0.001
BMI		
<18.5	0.96 (0.81, 1.13)	0.297
18.5–24.9	1.00	
25.0–29.9	1.07 (0.96, 1.20)	0.888
> = 30	1.02 (0.93, 1.17)	0.631
Range of eGFR		
> = 60	1.00	
45–59	1.06 (0.81, 1.39)	0.631
30–44	1.25 (0.95, 1.65)	0.102
<30	1.44 (1.08, 1.91)	0.004

We then looked at various clinical characteristics associated with individual malignancies (Table 3). African Americans formed a greater proportion of breast cancer patients (20.6%), as compared to other malignancies. Diabetes was more prevalent in patients with gynecologic malignancies (55.9%), as compared to patients with hematologic malignancies (29.4%). Over 60% of patients with thoracic malignancies had a history of smoking compared to 32.3 percent of patients with gynecologic malignancies. Patients with eGFR less than 30 mL/min comprised a higher proportion of patients with hematologic malignancies (20.6%).

**Table 3:** Patient characteristic summary by cancer categories.

	Breast	GI	Gynecologic	Head neck	Hematologic	Prostate	Thoracic	Urinary tract	P value
Median (IQR) age at cancer	71 (64, 79)	69 (61, 78)	68 (60, 75)	67 (58, 76)	70 (55, 77)	74 (67, 79)	71 (64, 79)	69 (60,78)	<0.001
Male (%)	0	59	0	67	66	100	28	72	<0.001
Race (%)									<0.001
White	74.8	78.6	81.8	79.8	94.2	81.0	81.2	84.6	
AA Black	20.6	13.8	13.9	13.9	2.9	13.2	12.7	9.8	
Hispanic	1.3	2.5	1.1	1.6	2.9	2.7	2.0	2.3	
Others	3.4	5.1	3.2	4.7	0	3.1	4.1	3.4	
Diabetes (%)	47.9	49.3	55.9	39.5	29.4	45.2	48.0	44.3	<0.001
Hypertension (%)	100	99.0	98.8	99.2	100	99.8	99.2	98.9	<0.001
CAD (%)	25.2	36.1	30.1	35.6	29.4	43.4	41.5	42.7	<0.001
Smoking history (%)	40.8	53.8	32.3	52.7	35.3	53.4	60.9	54.9	<0.001
BMI (%)									<0.001
<18.5	1.3	7.5	5.4	7.8	11.9	8.1	9.7	5.7	
18.5–24.9	12.7	23.9	11.9	17.9	26.9	17.5	26.5	18.9	
25.0–29.9	28.3	29.9	16.4	35.9	35.8	37.5	25.5	30.1	
> = 30	57.7	38.6	66.3	38.2	25.4	36.9	38.3	45.3	
Range of eGFR									<0.001
> = 60	2.1	1.8	2.2	3.8	1.5	2.7	2.2	1.1	
45–59	68.5	60.7	64.5	60.5	54.4	66.1	60.1	55.3	
30–44	18.9	23.8	20.4	17.8	23.5	16.3	19.8	25.4	
<30	10.5	13.6	12.9	17.8	20.6	14.9	17.9	18.3	

On multivariate analysis, the factors associated with increased mortality in patients who developed cancer included: increasing age (HR: 1.03 for each year; 95% CI: 1.02, 1.04), presence of diabetes (HR: 1.18: 95% CI 1.02, 1.37) and lower eGFR. Patients with eGFR less than 30 ml/min/1.73m^2^ had the highest risk of developing cancer (HR: 2.40; 95% CI 1.21, 4.72). Interestingly, patients with BMI ≥25 had decreased mortality compared to those with BMI between 18.5 and 24.9. (Table 4).

**Table 4:** Multivariable survival analysis of factors affecting mortality after cancer diagnosis.

	Hazard Ratio (95% CI)	P value
Age at cancer per year	1.03 (1.02, 1.04)	<0.001
Male	1.01 (0.82, 1.16)	0.930
Race		
White	1.00	
AA Black	1.12 (0.91, 1.37)	0.274
Hispanic	1.14 (0.71, 1.82)	0.597
Others	1.37 (0.98, 1.88)	0.052
Diabetes	1.18 (1.02, 1.37)	0.022
CAD	1.12 (0.97, 1.29)	0.109
Smoking history	1.27 (1.10, 1.47)	<0.001
BMI		
<18.5	1.05 (0.80, 1.37)	0.725
18.5–24.9	1.00	
25.0–29.9	0.81 (0.67, 0.97)	0.022
> = 30	0.75 (0.62, 0.91)	0.002
Range of eGFR		
≥60	1.00	
45–59	1.57 (0.81, 3.04)	0.180
30–44	2.22 (1.14, 4.34)	0.019
<30	2.40 (1.21, 4.72)	0.011

A multivariate survival analysis regression was performed for factors associated with mortality in breast cancer, gastrointestinal cancer, prostate cancer, thoracic malignancies and urinary tract malignancies. The other groups of malignancies were excluded due to small numbers. Increasing age at cancer diagnosis was associated with worse overall survival on multivariate analysis for gastrointestinal, prostatic, thoracic and urinary tract malignancies (Table 5). Race and ethnicity were not associated with survival except in thoracic malignancies where Hispanics had a higher risk of death (HR; 3.68; 95% CI 1.55, 8.71). Patients with diabetes had an increased risk of death from prostate cancer (HR: 1.53; 95% CI 1.06, 2.19), but not other malignancies. Similarly, a history of smoking was associated with increased mortality from thoracic malignancies (HR: 1.61; 95% CI 1.13, 2.30). Coronary artery disease, BMI and baseline eGFR were not significantly associated with a risk of death in any of the malignancies studied.

**Table 5:** Multivariable survival regression of factors affecting mortality in individual cancers.

Hazard Ratio (95% CI)					
	Breast	Gastrointestinal	Prostate	Thoracic	Urinary tract
Age at cancer per year	1.00 (0.98, 1.04)	1.03 (1.02, 1.04)	1.08 (1.06, 1.10)	1.04 (1.02, 1.05)	1.03 (1.02, 1.05)
Male	NA	1.28 (0.97, 1.64)	NA	1.43 (1.00, 2.03)	1.02 (0.71, 1.46)
Race					
Caucasian	1.00	1.00	1.00	1.00	1.00
AA	1.33 (0.69, 2.54)	0.84 (0.55, 1.27)	1.21 (0.73, 2.01)	1.23 (0.80, 1.89)	1.70 (1.05, 2.75)
Hispanic	1.00 (0.71, 1.32)	1.22 (0.53, 2.79)	0.60 (0.18, 2.00)	3.68 (1.55, 8.71)	0.81 (0.20, 3.31)
Others	1.81 (0.87, 3.08)	0.79 (0.46, 1.37)	1.61 (0.64, 4.03)	0.67 (0.27, 1.66)	2.93 (1.43, 5.99)
Diabetes	1.64 (0.86, 3.09)	1.30 (0.99,1.71)	1.53 (1.06, 2.19)	1.29 (0.93, 1.78)	1.13 (0.80, 1.55)
CAD	1.41 (0.76, 2.61)	0.89 (0.67, 1.19)	1.11 (0.78, 1.56)	1.21 (0.89, 1.65)	1.35 (0.98, 1.87)
Smoking history	1.57 (0.88, 2.78)	1.13 (0.87, 1.48)	1.08 (0.76, 1.53)	1.61 (1.13, 2.30)	1.23 (0.88, 1.72)
BMI					
<18.5	NA	0.85 (0.51,1.40)	1.50 (0.81, 2.78))	1.17 (0.68, 2.00)	1.63 (0.85, 3.13)
18.5–24.9	1.00	1.00	1.00	1.00	1.00
25.0–29.9	0.54 (0.22, 1.31)	0.72 (0.51, 1.00)	0.91 (0.57, 1.44)	0.99 (0.66, 1.49)	0.96 (0.61, 1.52)
> = 30	0.64 (0.30, 1.37)	0.66 (0.47, 7.92)	0.75 (0.43, 1.26)	0.79 (0.53, 1.19)	0.88 (0.57, 1.34)
Range of eGFR					
> = 60	1.00	1.00	1.00	1.00	1.00
45–59	NA	1.93 (0.47, 7.91)	1.20 (0.29, 4.93)	0.96 (0.35, 2.65)	NA
30–44	NA	2.97 (0.72, 12.30)	1.74 (0.41, 7.43)	1.48 (0.52, 4.22)	NA
<30	NA	3.82 (0.90, 16.18)	1.92 (0.44, 8.40)	1.75 (0.60, 5.06)	NA

## Discussion

This is a large single institutional study, evaluating the risk of malignancy and outcomes from selected cancers in patients with non-dialysis dependent chronic kidney disease. In this cohort of patients followed over a 20-year period, 20% developed an invasive malignancy. Increasing age, male sex, and lower eGFR were associated with an increased risk of developing malignancy.

Chronic kidney disease seems to be a risk factor for the development of malignancy. Almost 1 in 5 (22%) patients diagnosed with CKD between the ages of 50 and 80 years developed a malignancy in the present analysis. This number is higher than the reported lifetime risk of cancer in the general population in this age group. The lifetime risk of developing a cancer is one in 16 in both males and females between the ages of 50 and 60 years, while the risk in patients between the ages of 60 and 69 years is 1 in 7 in males and 1 in 10 in females [17]. These findings are similar to those found in recent analysis of non-dialysis dependent Chinese CKD patients that showed an increased risk of the cancer in patients with eGFR < 60 ml/min//1.73m^2^ (HR: 2.08; 95% CI 1.22–3.53). [18]. Similarly, another Korean study found that the overall incidence of cancer in patients with CKD was >1000/100,000 person-years [19]. A third study from the United Kingdom also found that a lower GFR was associated with an increased risk of incident cancer [20]. In contrast, other studies have not found an increased risk of cancer with lower eGFR [7, 21]. One possible explanation for this association may be that since CKD patients are monitored more closely, cancer may be detected more often in these patients leading to a detection bias.

The mechanisms underlying the increased risk of cancer in this cohort of patients are unclear. However various plausible theories have been proposed. Patients with reduced GFR appear to have an inflammatory pro-oxidant microenvironment, which may promote carcinogenesis [22]. Immune dysfunction, which is thought to increase cancer risk, seems to be prevalent in patients with CKD [23]. Lastly, uremic toxicity and the accumulation of compounds such as indoxyl sulfate and p-cresyl sulfate, may be associated with impaired DNA repair possibly promoting carcinogenesis [24].

We found that a history of smoking was associated with development of malignancy. Interestingly, diabetes was associated with a slightly decreased risk of developing cancer in this patient population. This is a surprising finding since multiple previous studies have shown that diabetes is associated with an increased risk of developing cancer. There are many possible pathways that have been proposed to connect diabetes and cancer, including an increase in insulin/IGF-1 signaling, lipid and glucose uptake and metabolism, alterations in the profile of cytokines and chemokines [25]. One possible explanation for this anomaly may be the role of IGF-1. Studies have shown that IGF-1 levels are lower in the serum and liver but increased in the kidneys in patients with diabetes [26]. At the same time, serum levels are normal in patients with chronic kidney disease. It is possible that this complex interaction between IGF-1 levels in various organs in diabetes and chronic kidney disease may explain our findings. Despite the decreased risk of developing cancer in patients with diabetes, diabetes was associated with increased mortality in patients who developed cancer.

We found that increasing age, diabetes, smoking history and lower eGFR at diagnosis were associated with increased mortality in patients with cancer. In a single center study from Japan, the presence of CKD at the time of stage IV cancer diagnosis was associated with increased mortality [27]. Similar findings were seen in an Australian study which showed increased cancer mortality in patients with an estimated glomerular filtration rate < 60 mL/min/1.73 m^2^ [28]. A large study from the UK also suggested that an eGFR <90 ml/min/1.73m^2^ was associated with increased cancer mortality [20]. This is not particularly surprising, since treatment modification may commonly be needed in patients with decreased eGFR to decrease toxicity of chemotherapy agents. When we evaluated factors associated with mortality in the most common cancers in our cohort, lower eGFR was not associated with an increased risk of mortality in any of these cancer subtypes. Patients with eGFR <30 ml/min/1.73 m^2^ had a higher risk of death as those patients with eGFR greater than 60 ml/min/1.73 m^2^, but these differences were not statistically significant. In the aforementioned Australian study, the only cancers where progressive CKD was associated with increased mortality were breast cancer and urinary tract malignancies [28]. Unfortunately, we were not specifically able to look at these 2 types of cancers specifically in relation to various glomerular filtration rate levels due to the small number of patients.

The main limitation of this analysis is its retrospective nature. Another limitation is that it is a single institution study and hence these findings will need to be confirmed in other settings. However, given the large cohort of patients and the long follow-up duration, these findings appear to be robust. We were not able to examine the interaction of hypertension and its effect on either development of malignancy or outcomes from cancer, because almost all our patients had a history of hypertension. Alcohol was not included as a covariate given the lack of specific information regarding alcohol usage in this cohort. Also, smoking status, BMI and kidney function were registered only at baseline. These parameters could have potentially changed during the follow-up period, but we did not address the changes as we wanted to assess the risk of cancer development at a given degree of kidney function.

This study utilizes EHR data as represented in a clinical data warehouse (CDW). CDW’s leverage national and international controlled medical terminology data standards to manage these data elements. This requires analysts to understand multiple coding systems based on clinical domains. The use of ICD-9/10 systems are common in epidemiology analyses. Use of ICD codes in the EHR systems varies in the US domain. It is largely a system leveraged for reimbursement and is coarse in nature. SNOMED CT is far more granular and robust for analyses. Further, the Office of the National Coordinator supports its use in problem list management. The use of two distinct coding systems for patient diagnoses presented some problems for initial data analysis. This was rectified by mapping ICD9/10 codes and SNOMED CT concepts into homogenized diagnostic groupings. Members of the CRANE development team and medical terminologists participated in this study. As a result, the use of multiple medical terminologies was only a minor inconvenience overall, it does highlight an overall need to expand general understanding of EHR data if such data are to be leveraged effectively.

In summary, chronic kidney disease is an increased risk factor for the development of various malignancies. The mechanisms for this increased risk should be investigated further. More importantly, age-appropriate cancer screening should be aggressively pursued in patients with progressive CKD.

## Supporting information

S1 File ICD-9-CM, ICD-10-CM, SNOMED CT inclusion codes, exclusion codes and codes for risk factors. (DOCX)
