# Supplementary material for: Risk of malignancy in patients with chronic kidney disease
Source: PLoS One. 2022 Aug 17;17(8):e0272910. doi: 10.1371/journal.pone.0272910 (PMC9385037; doi:10.1371/journal.pone.0272910)
Supplement: S1 File — (DOCX) [file pone.0272910.s001.docx]

**Supplemental Table 1. ICD-9-CM, ICD-10-CM, SNOMED CT Inclusion Codes**

| Condition | ICD-9-CM | ICD-10-CM | SNOMED CT |
| --- | --- | --- | --- |
| CKD | 285.21, 403, 404, 585, 585.1-5, 585.9 | D63.1, E08.22, E09.22, E10.22, E11.22, E13.22, I12.0, I12.9, I12, I13.0, I13.1, I13.10-11, I13.2, I13, N17-19, O10.2-10.23, O10.3-10.33 | <<709044004 |
|  | | | |
| ESRD | 403.01, 404.02-404.03, 585.6 | I12.0, I13.11, I13.2, N18.6 | <<46177005 |
| Cancer | 140-209, 357, 512, 790, 795-797 | C00-C96, O9A, R18.0, R53.0, R85.614, R87.614, R87.624, R97.1, Z08-09, Z40.0, Z85, V10, V71.1 | <<271323007, <<255056009, <<363506007, <<363429002, <<428905002, <<255077007, <<363493006, <<363358000, <<449096009, <<188361007, <<254837009, <<363514001, <<430556008, <<399068003, <<363517008, <<419052002, <<269475001, <<27524009, <<372130007, <<372062007 |

Patients were included by having CKD not related to ESRD, unless that ESRD was secondary to a cancer diagnosis.
Abbreviations: ICD-9-CM, International Classification of Diseases, Ninth Revision, Clinical Modification, ICD-10-CM, International Classification of Diseases, Tenth Revision, Clinical Modification, SNOMED CD, Systematized Nomenclature of Medicine -- Clinical Terms, CKD, chronic kidney disease, ESRD, end stage renal disease

**Supplemental Table 2. ICD-9-CM, ICD-10-CM, SNOMED CT Exclusion Codes**

| Condition | ICD-9-CM | ICD-10-CM | SNOMED CT |
| --- | --- | --- | --- |
| ESRD | 403.01, 404.02-404.03, 585.6 | I12.0, I13.11, I13.2, N18.6 | <<46177005 |
| Complication occurring during Pregnancy |  |  | <<609496007 |
| Kidney Transplant | 996.81, V42.0 | T86.1-86.13, T86.9, Z48.22, Z94.0 | <<737295003 |

Abbreviations: ICD-9-CM, International Classification of Diseases, Ninth Revision, Clinical Modification, ICD-10-CM, International Classification of Diseases, Tenth Revision, Clinical Modification, SNOMED CD, Systematized Nomenclature of Medicine -- Clinical Terms, ESRD, end stage renal disease

**Supplemental Table 3. ICD-9-CM, ICD-10-CM, SNOMED CT Codes for risk factors**

| Condition | ICD-9-CM | ICD-10-CM | SNOMED CT |
| --- | --- | --- | --- |
| Hypertension | 401, 405, 572.3, 796.2, 997.91 | G93.2, H40.05, I10, I15, I27, I97.3, R03.0 | <<38341003 |
| Diabetes | 249-250, 253.5, 357.2, 588.1 | E08-E11, E13, E23.2, N15.1 | <<73211009 |
| COPD | 490-496.99 | J41-47 | <<13645005 |
| CAD | 410-414 | I21.01, I21.09, I21.11, I21.19, I25.1, I25.4-I25.9 | <<53741008 |

Abbreviations: ICD-9-CM, International Classification of Diseases, Ninth Revision, Clinical Modification, ICD-10-CM, International Classification of Diseases, Tenth Revision, Clinical Modification, SNOMED CD, Systematized Nomenclature of Medicine -- Clinical Terms, COPD, chronic obstructive pulmonary disease, CAD, coronary artery disease
